# Protein Kinase D1 Signaling in Angiogenic Gene Expression and VEGF-Mediated Angiogenesis

**DOI:** 10.3389/fcell.2016.00037

**Published:** 2016-05-04

**Authors:** Bin Ren

**Affiliations:** ^1^Department of Medicine, Medical College of WisconsinMilwaukee, WI, USA; ^2^Blood Research Institute, Blood Center of WisconsinMilwaukee, WI, USA

**Keywords:** protein kinase D, CD36, VEGF, gene expression, transcription, endothelial cells, angiogenesis, arteriogenesis

## Abstract

Protein kinase D 1 (PKD-1) is a signaling kinase important in fundamental cell functions including migration, proliferation, and differentiation. PKD-1 is also a key regulator of gene expression and angiogenesis that is essential for cardiovascular development and tumor progression. Further understanding molecular aspects of PKD-1 signaling in the regulation of angiogenesis may have translational implications in obesity, cardiovascular disease, and cancer. The author will summarize and provide the insights into molecular mechanisms by which PKD-1 regulates transcriptional expression of angiogenic genes, focusing on the transcriptional regulation of CD36 by PKD-1-FoxO1 signaling axis along with the potential implications of this axis in arterial differentiation and morphogenesis. He will also discuss a new concept of dynamic balance between proangiogenic and antiangiogenic signaling in determining angiogenic switch, and stress how PKD-1 signaling regulates VEGF signaling-mediated angiogenesis.

## Introduction

The protein kinase PKD (*PRKD*) is a serine threonine kinase consisting of three isoforms PKD-1,-2, and -3 (Rykx et al., 2003; Evans et al., 2010). Different PKD family members have unique and non-redundant roles. PKD-1 is essential for normal embryogenesis while PKD-2 regulates the functions of mature peripheral lymphocytes during adaptive immune responses (Matthews et al., 2010) and PKD-3 modulates airway epithelial barrier formation, the growth of breast and prostate cancer cells and vesicle trafficking (Anderson et al., 2005; Chen et al., 2008; Huck et al., 2012, 2014). Initially, PKD-1 was classified to an atypical member of protein kinase C (PKC) family and known as PKCmu. However, this kinase presents a catalytic domain distantly related to Ca^2+^-regulated kinase and it was thus classified to the calcium/calmodulin-dependent protein kinase superfamily (Manning et al., 2002; Rozengurt et al., 2005).

PKD-1 mRNA is highly expressed in such tissues as the heart, the lungs, and the brain, as well as in a variety of cell types including vascular endothelial cells (VECs), fibroblasts, and dendritic cells. This indicates that PKD-1 is a key regulator in tissue homeostasis and cellular functions. PKD-1 mediates signaling pathways important in cardiovascular diseases, immune functions and cancer (Parra et al., 2005; Fielitz et al., 2008; Ha et al., 2008b; LaValle et al., 2010a,b; Wille et al., 2014). It may be involved in myocardial responses to ischemia and arterial remodeling (Avkiran et al., 2008; Ren et al., 2014, 2015). It also regulates cardiac energy homeostasis by influencing the secretion of cardiac lipoprotein lipase, an enzyme important in controlling heart metabolism in experimental diabetes (Wang and Rodrigues, 2015). The studies on lung microvascular ECs (MVECs) indicate that PKD-1 is required for PMA- and DAG-induced phosphorylation of myristoylated alanine-rich PKC substrate and hyperpermeability (Tinsley et al., 2004).

Interestingly, *PRKD1* is likely the gene targeted in clinical trials for schizophrenia drugs, implicating its functional significance in the brain functions. The authors suggested that bryostatin, a partial agonist at PKD, represents a promising drug for the treatment of schizophrenia (Lencz and Malhotra, 2015). GWAS studies show that *PRKD* gene is associated with body mass index (Speliotes et al., 2010; Comuzzie et al., 2012), suggesting the involvement of PKD-1 signaling in the pathobiology of diet-induced obesity (Huang et al., 2013; Dong et al., 2015; Yuan et al., 2015).

In the vascular system, PKD-triggered signaling pathways in ECs appear to process angiogenic information so that ECs respond appropriately to the environmental stimuli. This may be involved in VEGF-stimulated phospholipase Cγ1 (PLCγ1) signaling (Wong and Jin, 2005; Qin et al., 2006) and LPA-mediated transcriptional repression of CD36 (Ren et al., 2011). In this review, the author will briefly introduce the essential features of PKD-1 in structures and functions. Subsequently, he will discuss regulation of angiogenic gene expression by PKD-1 and its biological implications. He will specifically emphasize how CD36 transcription is regulated by PKD-1-FoxO1 signaling axis and what this axis implicates in arterial differentiation and morphogenesis. Finally, he will discuss a new concept of dynamic balance between proangiogenic and antiangiogenic signaling in determining angiogenic switch and focus on PKD-1 signaling in the regulation of VEGF-mediated EC functions and angiogenesis

## Structural features and general functions of PKD-1

**Figure 1:**
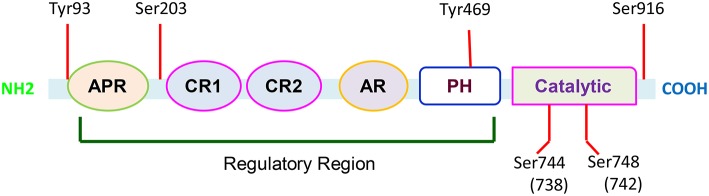
**Functional domains of PKD-1**. PKD-1 contains multiple functional domains including alanine and proline rich domain (APR); cysteine-rich, zinc finger-like domains (CR1 and CR2); acidic-rich region (AR); and pleckstrin homology domains (PH). There are six conserved phosphorylation sites. In response to a variety of biological factors, these sites can be phosphorylated to activate PKD-1 and regulate fundamental cellular functions.

PKD is encoded by the *PRKD1* gene that is located on human chromosome14q11. The gene transcript consists of 18 exons, and encodes a protein with 912 amino acid residues. PKD-1 contains a C-terminal kinase domain and a variable N-terminal regulatory domain with two highly conserved cysteine-rich zinc finger-like motifs (CR1 and CR2) and a pleckstrin homology (PH) domain inserted between the cysteine-rich motif and the catalytic domain. Different from protein kinase PKC, PKD contains PH domain within the regulatory region. The catalytic domain is distantly related to Ca^2+^-regulated kinase, and a highly hydrophobic stretch of amino acids is located in its N-terminal region (Lint et al., 2002; Rozengurt et al., 2005). The structural characteristics make this kinase unique in the regulation of cellular functions through activating its multiple phosphorylation sites (Figure 1).

PKD-1 remains inactive in cytosol partly through auto-inhibition of its catalytic activity by the PH domain. The multisite phosphorylations of the kinase control its signaling spatiotemporally. The phosphorylation in specific sites changes the catalytic activity and influences docking interactions with cellular scaffolds and trafficking to signaling microdomains in the subcellular compartments (Steinberg, 2012). G-protein subunits βγ directly interacts with the PH domain to activate the kinase by releasing the auto-inhibition of catalytic domain (Jamora et al., 1999; Waldron and Rozengurt, 2003). Once PKD-1 is stimulated by engaging G protein–coupled receptors (GPCRs) in response to growth factor signaling or oxidative stress, the molecules are able to be translocated to the plasma membrane. In the plasma membrane they are phosphorylated and activated. However, these molecules will ultimately be accumulated in the nucleus (Matthews et al., 2000) while its subcellular localization is regulated by the N-terminal domain, and the zinc finger-like motifs in particular (Rykx et al., 2003). The major catalytic sites are located at ser-744 and ser-748 or ser-738 and ser-742 within the activation loop of the catalytic domain, respectively in mouse and human PKD-1 (Iglesias et al., 1998) and in ser-916 (Matthews et al., 1999; Rozengurt et al., 2005). The phosphorylation activates PKD-1, whereas binding to 14-3-3 or chaperon protein p32 decreases its activity. Additionally, in response to apoptosis inducing agents, PKD-1 undergoes cleavage, releasing a 62 kD catalytic fragment by caspase 3 (Endo et al., 2000).

PKD-1 signaling functions downstream of PLCγ1, GPCRs, and tyrosine kinase receptors. The signaling activation phosphorylates downstream targets at specific sites, thereby regulating subcellular localization and/or its activity (Rozengurt, 2011) and the cellular processes such as DNA synthesis, proliferation, and invasion/migration (LaValle et al., 2010b). Importantly, PKD-1 signaling locates upstream of PI3K-Akt and MAPK/Erk1/2 signaling pathways (Guha et al., 2010) and is able to induce NF-kB activity in cells exposed to GPCR agonists or oxidative stress (Storz et al., 2005), which implicates the central roles of this kinase in EC functions and arteriogenesis (Ren et al., 2010, 2015; Tirziu et al., 2012).

## Regulation of transcriptional expression of angiogenic genes by PKD-1

Gene expression controls cellular phenotypes. Different cell types express characteristic sets of transcriptional regulators, thus controlling the expression of cellular specific genes through turning specific combinations of regulators on and off. The gene regulation drives the processes of cellular differentiation and morphogenesis to produce different cell types that possess varying gene expression profiles. The differential expression of angiogenic genes is critical in EC heterogeneity and angiogenesis (Adams and Alitalo, 2007; Aird, 2012; Ren, 2015; Yuan et al., 2016).

More and more studies show that PKD-1 signaling regulates the transcriptional expression of genes that are important in angiogenesis. The early growth response 3 (Egr3) is a member of a zinc-finger-like transcription factor subfamily. VEGF activates PKD-1 *via* the VEGFR2/KDR-PKC signaling, and subsequently induces Egr-dependent transcriptional activation and *Egr3* expression (Liu et al., 2008), whereas inhibition of *Egr3* expression decreases VEGF-mediated EC proliferation, migration and tubulogenesis (Liu et al., 2008). Recently, Zhao et al. showed that the calcium-PLC-PKC-PKD-1 pathway regulates VEGF-induced mRNA expression of TR3-TV2 and TR3-TV3 (Zhao et al., 2014). PKD-1/HDAC7/MEF2 signaling together with Erk1/2 pathway also regulates VEGF-induced Nur77 expression during VEGF-induced EC activation (Ismail et al., 2012).

### PKD-1 interaction with histone deacetylases to regulate angiogenic gene expression

Histone acetylation/deacetylation regulates transcriptional expression of genes through a dynamic balance between histone acetyltransferases and histone deacetylases (HDACs). HDACs are critical to inhibiting acetylation of nucleosome histones. Interestingly, HDAC5 and HDAC7, highly expressed in ECs (Mottet et al., 2007; Altschmied and Haendeler, 2008), are the substrate of PKD-1 (Avkiran et al., 2008) and regulate EC functions and angiogenesis (Wang et al., 2008; Urbich et al., 2009). VEGF stimulates HDAC5 phosphorylation and nuclear export in ECs *via* a VEGFR2-PLCγ-PKD-dependent pathway. Moreover, PKD-1 signaling interacts with HDAC5 to promote transcriptional activation of myocyte enhancer factor-2 (MEF2) and a specific subset of gene expression in response to VEGF including NR4A1, an orphan nuclear receptor involved in angiogenesis (Ha et al., 2008b). VEGF-mediated PKD-1 signaling also stimulates HDAC7 phosphorylation and cytoplasmic accumulation, thus modulating the expression of HDAC7-targeting and VEGF-response genes as well as VEGF-stimulated EC migration, tube formation, and sprouting angiogenesis (Mottet et al., 2007; Ha et al., 2008a). A similar mechanism is involved in the induction of PDGF-B/PDGFR-β expression and subsequent proangiogenic responses (Mottet et al., 2007). These studies indicate that PKD-1 interacts with specific HDACs to function as a molecular switch for controlling angiogenic gene transcription and VEGF-mediated angiogenesis.

### Regulation of arteriogenic gene expression by PKD-1 in microvascular endothelial cells

In primary MVECs, the lysophosphatidic acid (LPA), a lipid signaling mediator, regulates angiogenesis in a chicken chorioallantoic membrane assay and *in vivo* Matrigel assay (Rivera-Lopez et al., 2008; Ren et al., 2011) and promotes breast cancer angiogenesis in diet-induced obesity (Dong et al., 2015). LPA/PKD-1 signaling-mediated CD36 transcriptional repression is important in angiogenic processes (Ren et al., 2011). We found that PKD-1 signaling in MVECs promotes nuclear accumulation of HDAC7 in response to LPA (Ren et al., 2014), which is different from a previous report in HUVECs exposed to VEGF (Ha et al., 2008a). This indicates that a PKD-1 signaling “signature” is different in a specific cellular context. In certain cellular microenvironments, PKD-1 determines the formation of nuclear regulatory complex and aids in gene locus-specific nucleosomal enrichment of specific histone deacetylase (Fu and Rubin, 2011). PKD-1 may mediate HDAC7-FoxO1 interaction in the nucleus (Ren et al., 2014, 2015) and maintain context relevant EC functions *via* FoxO1-dependent regulation of CD36 transcription in HMVECs rather than *via* MEF-2-dependent regulation of matrix metalloprotease (Chang et al., 2006).

HDAC7 is an established regulator of chromatin structure and gene transcription (Haberland et al., 2009) and of angiogenesis (Chang et al., 2006; Mottet et al., 2007; Ha et al., 2008a). HDAC7-regulated gene repression and de-repression are indispensable for angiogenic functions (Ha et al., 2008a; Wang et al., 2008). In MVECs LPA/PKD-1 signaling appears to control the FoxO1 transcriptional switch (Hamik et al., 2006) *via* mediating the formation of a nuclear complex comprised of FoxO1 and HDAC7. PKD-1 signaling activation might specifically turn off the FoxO1 switch in the *CD36* gene locus for suppressing CD36 transcription, whereas arteriogenic gene reprogramming is initiated once *CD36* transcription is turned off in response to LPA (Ren et al., 2015). This may be associated with the proarteriogenic responses in a tumor microenvironment (Kohlenberg et al., 2013). These findings suggest that PKD-1 signaling epigenetically regulates arteriogenic gene transcription *via* modulation of chromatin remodeling and is involved in microvascular remodeling.

## PKD-1 functions in VEGF signaling and angiogenesis

### Basic concept of angiogenesis

Angiogenesis is a physiological or pathological process from which new blood vessels develop from pre-existing vessels, and in which ECs is the key (Carmeliet, 2000; Semenza, 2007; Ren, 2015). Physiological angiogenesis is fundamental for development, reproduction, and tissue repair, whereas pathological angiogenesis leads to aberrant neovascularization due to uncontrolled EC activity and contributes to ischemic cardiovascular disease, rheumatoid arthritis, and cancer (Carmeliet, 2000). The plaque angiogenesis is of functional significance in atherosclerosis (Moulton et al., 2003, 2004), and occurs more frequently in atheromas of patients with diabetes and unstable coronary syndromes (Burke et al., 1997). Antiangiogenic endostatin could thus control the progression of atherosclerosis by inhibiting plaque angiogenesis (Moulton et al., 1999; Ren et al., 2002, 2003).

Angiogenesis is controlled by biological and physical interactions between cells and extracellular matrices. Biological and mechanical signals integrate with other micro-environmental cues to control angiogenesis *via* a *dynamic signaling balance* between pro- and anti-angiogenic factors, while the dominant signaling determines the on and off of the angiogenic switch (Ren et al., 2006, 2009; Chen et al., 2013; Ren, 2015).

**Figure 2:**
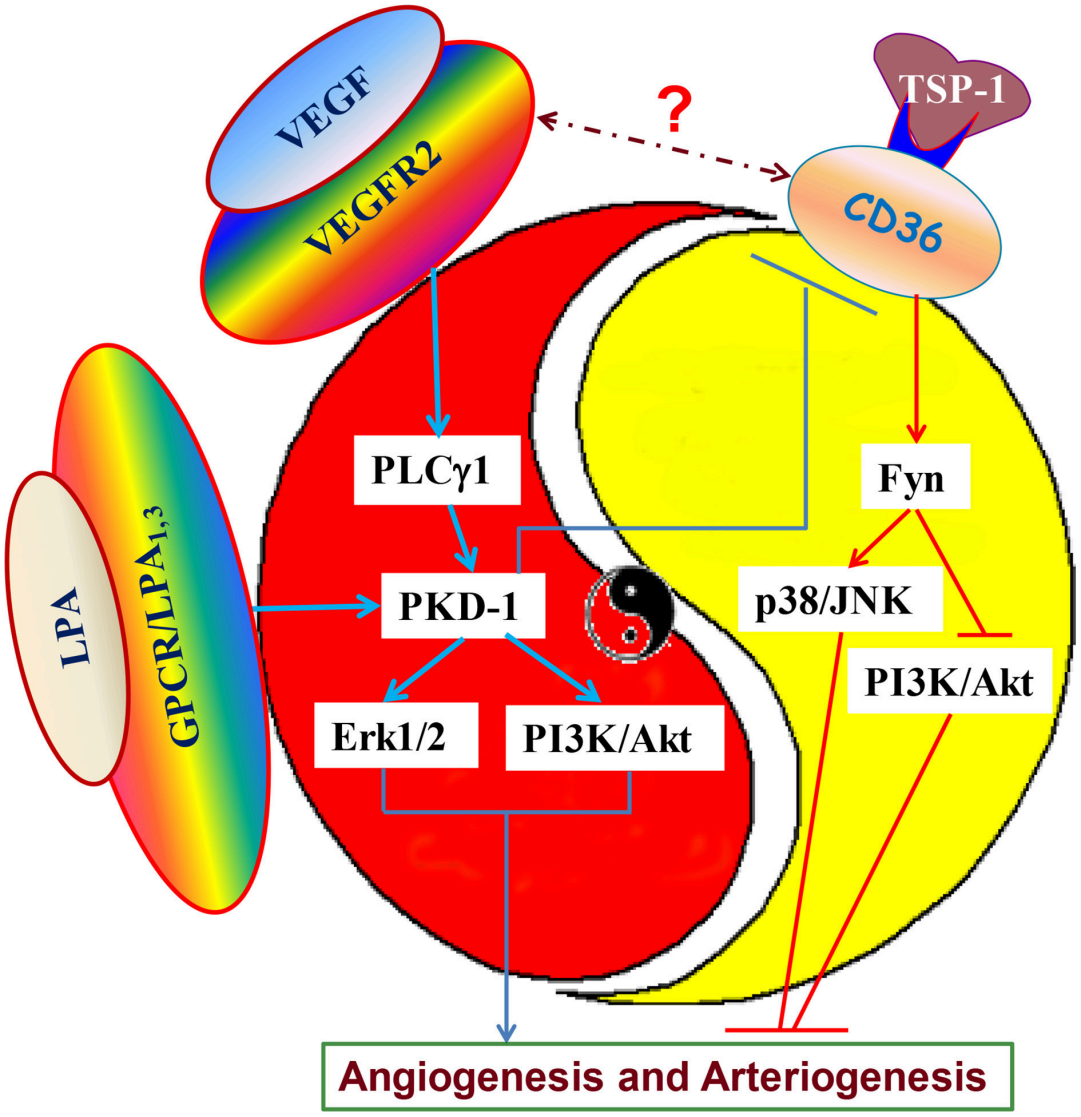
**Dynamic angiogenic signaling balance and angiogenic switch**. VEGF, as an important angiogenic cytokine, is critical for proangiogenesis, and proarteriogenesis via activating VEGF receptor 2. VEGF signaling via VEGF receptor 2 can activate PKD-1 pathway via PLCγ1 to stimulate MAPK/Erk1/2 and PI3K/Akt activation. However, oncogenes, such as ras and myc or tumor suppressor gene p53 down-regulate or up-regulate TSP-1 expression, and affect TSP-1 interaction with the CD36 receptor, altering the activities of Fyn, p38/JNK, and PI3K/Akt kinases. This interaction may produce disturbances in the dynamic signaling balance between angiogenesis stimulators and inhibitors. The dominant signaling will determine the angiogenic switch. LPA also activates PKD-1 signaling to suppress CD36 transcription to tip the signaling balance to VEGF-mediated proangiogenic and proarteriogenic responses. Inhibition of CD36 expression and its anti-angiogenic signaling can prime VEGF signaling for turning on the proangiogenic and proarteriogenic switch for angiogenesis and arteriogenesis.

Currently, the majority of the research focuses on pro- and anti-angiogenic factors to regulate angiogenic switch (Bergers and Benjamin, 2003). Actually, the *Yin and Yang* balance between pro- and anti-angiogenic signaling may control the angiogenic switch. This dynamic signaling balance could play a critical role in the regulation of pathophysiological and physiological angiogenesis. Moreover, the angiogenic receptors may serve as a pivotal axis to regulate this angiogenic process. The ligand and receptor interactions are the key to integrating signaling for the regulation of angiogenic switch. A good example is a *yin* and *yang balance* between antiangiogenic TSP-1 and proangiogenic VEGF signaling which determines angiogenic switch, and in which PKD-1 is a key player. PKD-1 functions downstream of VEGF/PLCγ1 signaling to stimulate angiogenesis. LPA, a lipid signaling mediator, may suppress CD36 expression *via* PKD-1 and regulate crosstalk between VEGFR2 and CD36, subsequently contributing to angiogenic switch (Figure 2). VEGF is essential for angiogenesis due to its regulation of biological responses in ECs during development and in disease, while TSP-1-CD36 signaling could crosstalk with VEGF signaling to control arteriogenic fate and angiogenic responses.

### PKD-1 in VEGF signaling-mediated EC functions

ECs is a key player in angiogenesis (Ren, 2015). VEGF signaling in ECs is regulated at multiple levels *via* three receptor tyrosine kinases or VEGF receptor 1-3 (VEGFR1-3). VEGF interactions with VEGFR2/KDR (kinase insert domain receptor) are critical for normal blood vessel development (Matsumoto and Claesson-Welsh, 2001; Folkman and Kalluri, 2004) and deregulates in pathological conditions (Wong et al., 2001; Ferrara et al., 2003). VEGFR-2-mediated VEGF signaling is also important in proangiogenic responses, including EC migration, proliferation and tubulogenesis, the key biological processes in angiogenesis (Wang, 2006; Evans et al., 2008).

PKD-1 signaling plays a key role in those processes in addition to its roles in regulation of EC survival, trafficking and immune response (Prigozhina and Waterman-Storer, 2004; Yeaman et al., 2004; Wang, 2006; Eiseler et al., 2007; von Wichert et al., 2008). VEGF activates PKD-1 *via* the VEGFR2/PLCγ/PKCα pathway, promoting Erk activation and DNA synthesis for EC proliferation (Wong and Jin, 2005). PKD-1 phosphorylation at tyrosine 463 by VEGF can activate PLCγ to stimulate proangiogenic responses (Qin et al., 2006) and activate Erk1/2 signaling (Kohlenberg et al., 2013), implicating its function in arterial differentiation (Lawson et al., 2003; Hong et al., 2006; Ren et al., 2015).

PKD-1 regulates collagen I-induced vascular morphogenesis *via* modulating GSK3β activity and integrin α(2)β(1) trafficking (Shin et al., 2012), whereas PKD-1-mediated integrin αvβ3 trafficking contributes to the angiogenic process by integrating and coordinating EC activity (di Blasio et al., 2010). These studies suggest that this kinase mediates the bidirectional communication between VEGFR and integrins. Furthermore, PKD-1 regulates VEGF-mediated host inflammatory responses and could lead to inflammatory angiogenesis. PKD-1 signaling is associated with VEGF-induced expression of proinflammatory cytokines *via* VEGFR2 including interleukin (IL)-6, CXC chemokines IL-8, and growth-related oncogene-alpha (GRO-alpha; Hao et al., 2009a). Additionally, PKD-1 interacts with heat shock proteins (HSPs) to regulate angiogenesis. PKC-mediated PKD-1 signaling regulates VEGF-induced HSP 27 phosphorylation at phosphorylation site serine 82 and tubulogenesis in HUVECs (Evans et al., 2008). Recently, PKD-1 was shown to activate endothelial nitric oxide synthase and orchestrate mammalian vascular tone through phosphorylation, concomitantly increasing NO synthesis. Inhibition of the kinase activity in mice abolishes VEGF-induced vasodilatation, indicating that PKD-1 is the key to transducing VEGF signaling for VEGF-induced vasodilatation (Aicart-Ramos et al., 2014). These studies suggest that PKD-1 signaling may regulate EC cross-talking with other cell types, such as vascular smooth muscle cells, *via* producing signaling molecules in the tissue microenvironments. Interestingly, the isoform PKD2 also regulates proliferation and migration in HUVECs, the two important process of angiogenesis, and this may be mediated by modulation of the expression of VEGFR-2 and fibroblast growth factor receptor-1 (Hao et al., 2009b).

### PKD signaling in tumor angiogenesis

Tumor angiogenesis is critical, not only in rapidly growing macroscopic tumors, but also in microscopic premalignant phase of neoplastic progression, and thus is considered as an integral hallmark of cancer (Hanahan and Weinberg, 2011). Emerging studies have begun to focus on PKD functions in tumor angiogenesis.

PKDs regulate both hypoxia-induced VEGF expression/secretion by the tumor cells and VEGF- stimulated angiogenesis, which are essential for the malignant progression of tumors. Studies show that PKD-1 promotes anchorage-independent growth, invasion, and angiogenesis in human pancreatic cancer including PDAC (Guha et al., 2010; Ochi et al., 2011). In a zebrafish/tumor xenograft model, this kinase is shown to promote angiogenesis and malignant progression (Hollenbach et al., 2013). Our study implicates that diet-induced obesity may promote tumor progression *via* LPA/PKD-1 signaling-mediated angiogenesis (Dong et al., 2015). Interestingly, PKD-1 signaling appears to regulate tumor angiogenesis and is implicated in tumor arteriogenesis as it may regulate CD36 expression and vascular remodeling in the tumor microenvionment (Kohlenberg et al., 2013).

The PKD-2 also mediates production of various angiogenic factors in human pancreatic cancer cells and stimulates the angiogenic response of the host vasculature (Azoitei et al., 2010). Moreover, this isoform regulates tumor cell communication with ECs in gastrointestinal tumors and glioblastomas and promotes tumor growth. Mechanistically, PKD-2 integrates signals from hypoxia and HSP90 pathways in the NF-κB/VEGF signaling axis to stimulate angiogenesis and malignant progression (Azoitei et al., 2014). PKD2 is also able to promote pancreatic cancer cell invasion in three-dimensional extracellular matrix cultures by stimulating expression and secretion of matrix metalloproteinases 7 and 9 (MMP7/9) whereas MMP9 stimulates PKD2-mediated tumor angiogenesis by releasing extracellular matrix-bound VEGF and increasing its bioavailability (Wille et al., 2014).

## Summary and prospective

PKD-1 regulates the expression of a variety of angiogenic genes. Interestingly, LPA/PKD-1 signaling suppresses CD36 transcription and reprograms MVECs for arteriogenic gene expression *via* a nuclear HDAC7-FoxO1 complex, implicating microvascular remodeling and tumor arteriogenesis (Kohlenberg et al., 2013; Ren et al., 2015). This not only indicates the plasticity of adult ECs but also suggests the role of PKD-1 in EC heterogeneity (Aird, 2012; Ren, 2015). The PKD-FoxO1 signaling axis may thus function as a molecular link for a dynamic balance between pro- and anti-angiogenic signaling by controlling EC CD36 transcription, possibly regulating arteriogenesis *via* this axis. Activation of this pathway *in vivo* may promote vascular remodeling and the functional stability of the arterial networks (Ren et al., 2016a). The precise mechanisms and functional consequences are worthy of future investigation.

Furthermore, PKD-1 signaling is essential for VEGF signaling-mediated angiogenic functions and is implicated in tumor arteriogenesis. VEGF signaling is regulated by the tight control of intracellular VEGFR2 localization (Bhattacharya et al., 2005; Lanahan et al., 2010). VEGF induces PKD-1 signaling and angiogenesis *via* regulation of αvβ3 integrin recycling (Woods et al., 2004; White et al., 2007; di Blasio et al., 2010). Therefore, PKD-1 signaling might regulate VEGFR-2 trafficking, an important process of VEGF signaling and of arterial morphogenesis (Lanahan et al., 2010; Simons, 2012) *via* cross-talking with integrin αvβ3 and CD36, subsequently turning on angiogenic switch for arterial differentiation of ECs to promote arteriogenesis. ECs is essential for developmental and adult arteriogenesis (Ren et al., 2010; Simons and Eichmann, 2015) and EC PKD-1 signaling is essential for improving tissue perfusion through proarteriogenic reprogramming in the ischemic conditions(Ren et al., 2016b). It is important to understand how PKD-1 signaling interacts with FoxO1 and histone deacetylases to initiate an epigenetic and transcriptional program and control arteriogenic gene transcription and morphogenesis. This understanding will provide insight into finding new and effective therapeutic targets and strategies against cardiovascular disease, cancer, and obesity.

## Author contributions

The author confirms being the sole contributor of this work and approved it for publication.

### Conflict of interest statement

The author declares that the research was conducted in the absence of any commercial or financial relationships that could be construed as a potential conflict of interest.
